# Legal Immigration Status is Associated with Depressive Symptoms among Latina Transgender Women in Washington, DC

**DOI:** 10.3390/ijerph15061246

**Published:** 2018-06-12

**Authors:** Thespina Yamanis, Mannat Malik, Ana María del Río-González, Andrea L. Wirtz, Erin Cooney, Maren Lujan, Ruby Corado, Tonia Poteat

**Affiliations:** 1 School of International Service, American University, 4400 Massachusetts Ave NW, Washington, DC 20016, USA; maren.lujan@gmail.com; 2 Bloomberg School of Public Health, Johns Hopkins University, Baltimore, MD 21205, USA; mmalik7@jhu.edu (M.M.); awirtz1@jhu.edu (A.L.W.); ecooney2@jhmi.edu (E.C.); tpoteat@jhu.edu (T.P.); 3 Department of Psychology, The George Washington University, 2121 I St NW, Washington, DC 20052, USA; amdelrio@gwmail.gwu.edu; 4 Casa Ruby, 7530 Georgia Ave NW, Washington, DC 20012, USA; corado@casaruby.org

**Keywords:** transgender women, immigrants, Latinas, immigration status, documentation status, depression, depressive symptoms

## Abstract

Latina transgender women (LTW) are disproportionately vulnerable to depression, although the role of immigration/documentation status (legal authority to live/work in the U.S.) in depression has not been explored. LTW in Washington, DC were recruited into a cross-sectional study via convenience sampling. Most were Spanish-speaking Central American immigrants. Participants completed rapid HIV tests, and a Spanish-language survey assessing recent depressive symptoms (PHQ-2), sociodemographics, and factors from the minority stress framework: structural stressors (documentation status, stable housing), social stressors (discrimination, fear of deportation, violence) and coping resources (social support, resilience). Among immigrant LTW (*n* = 38), 24 were undocumented. Among the undocumented, the average PHQ-2 score was 2.7, and among the documented, the average PHQ-2 score was 1.4 (*p* < 0.05). Undocumented LTW were significantly more likely to experience employment discrimination, recent unstable housing, and fear of deportation. Bivariate and multiple linear regressions were performed to assess the relationship between documentation status and other correlates of past two week depressive symptoms. In multivariate analysis, PHQ-2 scores were inversely associated with being documented (*p* < 0.01), having an income above the federal poverty level, higher friends’ social support, and increased resiliency. Documentation status is an important correlate of depressive symptoms among LTW that should be considered within the context of health interventions.

## 1. Introduction

Transgender people living in the U.S. experience disproportionately elevated rates of depression, with prevalence as high as 44.1% [1] to 62.0% [2]. A recent systematic review on the mental health of transgender and gender non-conforming people found consistently high levels of depressive symptoms across studies [3]. The review was guided by the minority stress framework, which posits that social and environmental stressors disproportionately impact sexual and gender minority populations because of intersecting identity-based oppression; these stressors, in turn, cause poor mental health [4,5]. Theoretically, these stressors can occur across levels of the socioecological framework, from distal (e.g., structural and institutional polices) to proximal (e.g., internal coping) [4]. Studies demonstrate that risk factors for mental health outcomes among transgender and gender non-conforming populations include social processes such as discrimination and interpersonal violence [3]. Protective factors for mental health include social support, community connectedness, and coping [3].

Latina transgender women (LTW) constitute a multiply marginalized group for whom mental health is a significant public health issue. One study found that LTW reported significantly higher depressive symptoms compared to African-American, White, and Asian/Pacific Islander transgender women [6]. In a 2001 study of 220 immigrant LTW living in Los Angeles (LA), California, 35% reported significant depressive symptoms (PHQ-9 ≥ 15) [7]. In a 2012 study, 64.2% of a sample of 110 mostly foreign-born LTW living in San Francisco (SF)/Oakland, California reported clinically significant depressive symptoms (CES-D ≥ 16) [6]. Consistent with the minority stress framework, across studies, LTW’s rates of depressive symptoms were positively associated with racial/ethnic and sexual identity discrimination [7,8] and negatively associated with social support [8].

While the aforementioned studies among LTW included immigrants (those born outside of the U.S.), few explicitly explored immigration/documentation status (i.e., legal authorization to live/work in the U.S.) as a stressor that contributes to depression. In the LA study, half of the LTW population was undocumented (lacked legal authorization to live/work in the U.S.), and immigration status was not significantly associated with depressive symptoms [7]. Most of the sample was born in Mexico and lived in the U.S. for at least five years, suggesting that the sample may have had moderate to high levels of acculturation to the U.S. The SF/Oakland study included mostly foreign-born LTW and they reported significantly higher unmet need for social support, compared to African-American, White, and Asian/Pacific Islander transgender women [6]. The authors’ rationale for this finding included LTW’s loss of extended family following immigration, although family networks were not measured in the study [6].

Immigration/documentation status is a structural determinant of health that fits with the concept of a general stressor in the minority stress framework [4]. Research demonstrates that lack of legal status is an everyday stressor for immigrant Latinx (gender-neutral, all-inclusive plural term used in the U.S. to describe “Latinos”) that is linked to anxiety and depression [9]. The stress generated by a combination of lack of legal authorization to live/work in the U.S., anti-immigrant sentiment, and barriers to health and social services adversely impacts mental health for immigrant Latinx [10,11,12]. On the other hand, having legal authorization to live/work in the U.S. may protect mental health. In previous qualitative research, immigrant LTW who received legal asylum, which confers the right to live and work in the U.S. [13,14,15], reported improved mental health, more stable housing situations, and leaving abusive partners [16].

In this article, we conceptualized minority stressors across several levels, exploring their relationships with depressive symptoms among immigrant LTW living in Washington, District of Columbia (DC). Acknowledging prior research among LTW [16], we conceptualized legal immigration/documentation status and stable housing as structural stressors associated with depressive symptoms. We hypothesized that those immigrant LTW who were documented (i.e., received immigration relief or held a green card), had fewer depressive symptoms compared to immigrant LTW who were undocumented (i.e., did not receive immigration relief or legal authorization to live/work in the U.S.). In terms of social stressors, we conceptualized violence and discrimination as risk factors for depressive symptoms. Further, we investigated fear of deportation as a social stressor and risk factor for depressive symptoms among LTW using a new measure for fear of deportation. Finally, in terms of coping resources, we conceptualized social support and individual resilience as factors that protect against depressive symptoms.

## 2. Materials and Methods

### 2.1. Setting

Washington, DC has one of the highest proportions of transgender women in the U.S. at 2.8% [17]. The DC metropolitan area has a large share of U.S. undocumented immigrants, with continued growth despite declining national trends. The majority of these undocumented immigrants are from El Salvador, followed by other Central American countries [18]. The closest Federal Immigration Court is in Arlington, Virginia. In 2016, the Arlington Court had an asylum grant rate of 62% (compared to 2% in Atlanta, Georgia; 26% in Tucson, Arizona; and 74% in SF, California) [19].

### 2.2. STROBE Study

Between February and May 2017, LTW participated in the STROBE (Supporting Transgender Research and Opportunities in the Baltimore and DC Environments) study. The STROBE study aimed to: (1) describe the structural, social, and individual HIV risk and protective factors among transgender women of color in Baltimore, MD, USA and Washington, DC, USA; (2) estimate the prevalence of HIV within this population; and (3) assess knowledge and acceptability of potential HIV prevention interventions. The STROBE study started in Baltimore and expanded to DC to increase recruitment of transgender women of color. Because of the sizable population of LTW in DC and the presence of two community-based organizations (CBOs) that serve LTW, the STROBE survey was translated from English to Spanish to improve accessibility to LTW. A professional translator, who is also Central American, translated the survey. The Spanish survey was then reviewed in-depth by a Spanish-speaking LTW from Central America who provides social services to LTW in DC. She answered all the survey questions herself and provided feedback on language that would be understandable to her community. After incorporating her feedback, two other native Spanish speakers who work with the LTW community in DC reviewed the survey to be sure that all items were clear. The reading level of the survey was 7th grade.

Participants completed an interviewer-administered quantitative survey in English or Spanish on a tablet device, followed by rapid oral HIV testing (OraQuick Rapid HIV-1/2 test). They were not required to stay for the results of their test; nearly all participants who tested positive already knew their status. One participant who tested positive had not previously known her status and she stayed to receive the results. All participants who tested positive for HIV and stayed were offered linkage to confirmatory testing at local clinics. Interviewers used local resource guides to facilitate participants’ linkage to confirmatory HIV testing and referral to other health and social services when requested.

Study visits lasted between 60 and 90 minutes. Data collection took place in private rooms at well-known CBOs and community health centers, all located in areas easily accessible by public transportation. The study interviewer was a native Spanish speaker who worked on previous studies with the local Latinx sexual and gender minority community. Thus, she was a known and trusted person to community gatekeepers. On this study, she worked closely with CBO staff to advertise and recruit for the study. Participants received a $50 Visa gift card as an incentive.

### 2.3. Recruitment

LTW were recruited for the study through several convenience sampling approaches, suggested during formative qualitative research for the STROBE study (unpublished data). The study data collectors coordinated recruitment at local HIV and LGBTQ or transgender events, venues, and activities (e.g., National Transgender HIV Testing Day event, support groups for transgender women, community meetings). Flyers and information were distributed at community health centers and trusted CBOs, including two DC CBOs that primarily serve LTW.

### 2.4. Participants

A two-step method was used to identify transgender participants, including those who may not use the term *transgender* to describe their gender [20]. This method is widely recommended in the U.S., and measures (1) sex-assigned-at-birth and (2) current gender identity. Individuals are enrolled as transgender for the purposes of research when their responses to steps 1 and 2 are discordant.

Individuals were eligible to participate in the study if they met the following criteria: (1) 15 years old or older; (2) currently reside in the Baltimore or Washington, DC metropolitan areas; (3) assigned male sex-at-birth; (4) currently identify as a gender that is different from sex-assigned-at-birth (e.g., woman, transgender woman, gender non-binary, gender queer); (5) self-identified non-White racial identity (note: individuals who identified as White and another race were eligible to participate), (6) willingness to complete a rapid oral HIV test; and (7) speak English or Spanish. There were a total of 201 participants in the STROBE study.

This sub-analysis focused on participants who self-identified as Latina/Hispanic (of any race), were immigrants (born outside of the U.S.), and were not U.S. citizens. Immigrant LTW who met these criteria (*n* = 38) were all DC residents, age 18 or over, and completed the survey in Spanish.

### 2.5. Ethics

All participants gave verbal informed consent in Spanish before completing the study visit. The study protocol was approved by the Johns Hopkins School of Public Health Institutional Review Board (IRB00006279).

### 2.6. Measures

#### 2.6.1. Demographics

Demographic characteristics included age, sexual orientation, and race/ethnicity. Latina/Hispanic identity was presented as a separate question (“Do you identify as Latina/Hispanic?”) from racial identity, although many Latina/Hispanic participants indicated Latina/Hispanic race by typing it into the “other” race option. Socioeconomic factors, including income (responses collapsed into above vs. below federal poverty level), recent hunger (“In the past 30 days, how often did you go to sleep hungry because you didn’t have enough food?”), highest level of education, employment status (responses collapsed into employed vs. unemployed), and health insurance were also assessed.

#### 2.6.2. Immigration Experiences

Participants were asked if they entered the U.S. on a green card. Participants who reported entering without a green card were asked if they had ever applied for legal relief, for what type of relief they applied (e.g., U-Visa (applicants must be victims of certain crimes committed in the U.S. and must demonstrate their willingness to cooperate with law enforcement), asylum, temporary protected status), who their legal provider was, and whether the legal outcome was successful or still in progress. Participants who had never applied for legal immigration relief were asked for the reasons they had not applied. All participants were asked whether they were ever deported or detained. If they were detained, they were asked if they had ever experienced abuse by another detainee or a guard while in detention.

#### 2.6.3. Depressive Symptoms

Depressive symptoms during the past two weeks were assessed using the two-item Patient Health Questionnaire-2 (PHQ-2). Participants were asked how often, over the past two weeks, they had been bothered by: (1) having “Little interest or pleasure in doing things” and (2) “Feeling down or depressed or hopeless” [21]. Response options were not at all (0), several days (1), more than half the days (2), and nearly every day (3). The PHQ-2 is used as a screener for a recent major depressive episode [22]. Participants’ PHQ-2 sum scores (range: 0–6) were used as a continuous variable. For descriptive statistics, participants who scored a total of three or higher were categorized as having experienced recent depression [21].

To assess suicidal ideation, all participants were asked if they had ever had thoughts of killing themselves (response options: no, once, or more than once). Affirmative responses were collapsed to create a dichotomous suicidal ideation variable (lifetime suicidal ideation vs. none). Participants who reported any suicidal ideation in their lifetime were also asked if they had ever made a suicide attempt (defined as: purposefully hurt yourself with at least some intention to die).

#### 2.6.4. Alcohol and Drug Use

The three-item AUDIT-C screener was used to identify participants who are hazardous drinkers and/or have an active alcohol use disorder, based on drinking behaviors in the past twelve months [23]. The items assess drinking frequency (“How often do you have a drink containing alcohol?”), typical number of drinks (“How many standard drinks containing alcohol do you have on a typical day when you’re drinking?”), and frequency of binge drinking (“How often do you have six or more drinks on one occasion?”). Participants were classified as hazardous drinkers if their sum score was three or higher, based on the validated cutoff for cisgender (non-transgender) women [24].

Participants were first asked if they ever used drugs recreationally. Those who responded affirmatively received follow-up questions about use of non-injection and injection drugs in the past twelve months. They were shown a comprehensive list of substances including: heroin, cocaine, ecstasy or other club drugs, marijuana, pain pills, poppers, and methadone and/or suboxone (buprenorphine) prescribed for someone else, among others. Responses were collapsed into three mutually exclusive categories for illicit drug use in the past twelve months: no drug use, marijuana use only, and illicit drug use (excluding marijuana use). Possession of small amounts of marijuana is legal in DC [25].

#### 2.6.5. HIV Status and HIV Risk Behavior

Following the survey, HIV status was determined through rapid HIV antibody testing with oral fluid collection using the OraQuick *ADVANCE*^®^ Rapid HIV-1/2 Antibody Test. The test is highly accurate, demonstrating >99% sensitivity and specificity in clinical testing. Participants were also asked to self-report the result of their most recent HIV test.

Lifetime history of exchange sex was assessed through a series of questions about whether participants had ever exchanged sex for money, material goods, food, a place to sleep, drugs, hormones, or surgery. An affirmative response to any of these questions was categorized as having a history of exchange sex.

#### 2.6.6. Structural Stressors

**Figure 1:**
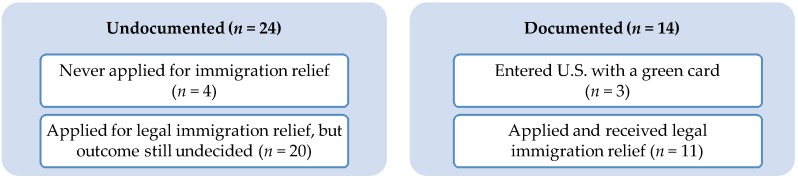
Current Documentation Status among Immigrant Latina Transgender Women (*n* = 38) in the STROBE Study, Washington, DC, USA, 2017.

Immigration/documentation status was a variable created based on participants’ responses to questions about their applications for immigration relief and whether they had a green card. Figure 1 illustrates the sample of LTW, divided into undocumented and documented groups.

Housing instability in the past twelve months was also assessed. Unstable housing experiences included “I moved to escape violence”; “I became homeless”; “I was forced to stay in a shelter that did not match my gender identity”; “I had to find different places to sleep for short periods of time, such as on a friend’s couch”; and “I had sex with people to stay in their homes.” Those participants who selected one or more of these experiences were categorized as unstably housed in the past twelve months.

#### 2.6.7. Social Stressors

Participants’ experiences of employment discrimination were assessed. Those who endorsed the question “Were you fired from your job or denied a job or promotion?” were categorized as having experienced employment discrimination.

Fear of deportation was assessed with a sixteen-item scale developed in Spanish for another study among Latino men who have sex with men (MSM) in DC [26]. The items were generated from qualitative work on fear of deportation among Spanish-speaking immigrant Latino MSM. The items described activities they avoided due to fear of deportation. Example items included: “Due to fear of deportation, how often do you avoid driving?”; “Due to fear of deportation, how often do you avoid calling attention to yourself? “Due to fear of deportation, how often do you avoid seeking healthcare even when you’re sick?” Response options were frequently (1), sometimes (2), rarely (3), never (4). Items were reverse-coded such that higher scores indicated greater fear. The scale reliability was high (α = 0.92) among this sample. The range of scale scores was 16–64, with a mean of 37.4 (std. dev. = 14.2). 

Lifetime and recent psychological, physical, and sexual violence experiences were assessed using a modified version of the Revised Conflict Tactics Scale (CTS-2) [27]. The CTS-2 is a measure of intimate partner violence that assesses a range of tactics used to perpetrate violence. We built on these measures of violence tactics, but expanded the questions to include identification of other perpetrators of violence and the period in which violence occurred (past twelve months or lifetime). Perpetrators included current partner, ex-partner, sex work client, family member, employer/co-worker, someone in the transgender community, someone in your neighborhood, police/law enforcement, stranger and other. Participants were queried about several violence experiences under each of the three violence types, psychological (example item: “Belittled or humiliated you in front of other people”); physical (example item: “Hit you with a fist or something else that could hurt you”); and sexual (example item: “Physically forced you to have sexual intercourse when you did not want to”). Those who responded “yes” to any of the violence experience statements in their lifetime received a follow-up question to assess whether that form of victimization occurred in the past twelve months. Three dichotomous, composite variables were created for having experienced any lifetime psychological, physical, and sexual violence; recent violence was also dichotomized for each violence type.

#### 2.6.8. Coping Resources

The survey included a twelve-item measure of perceived social support, which consists of three sub-scales: (four-items each): (1) family support (example item: “I can talk about my problems with my family”; α = 0.89); (2) support from friends (example item: “My friends really try to help me”; α = 0.87); and (3) support from significant others (example item: “There is a special person who is around when I am in need”; α = 0.88) [28]. Responses were scored on a four-point scale, ranging from strongly disagree to strongly agree. An overall summary score and sub-scale summary scores were calculated for each participant.

To assess individual resilience, participants responded to the six-item Brief Resilience Scale (example items include: “I tend to bounce back quickly after hard times”; “I have a hard time making it through stressful events”; α = 0.79) [29]. Responses were scored on a four-point scale, ranging from “strongly disagree” to “strongly agree.” A total mean score was calculated for each participant.

#### 2.6.9. Data Analysis

The documented and undocumented groups were compared using chi-square tests and independent samples t-tests according to demographic, immigration, alcohol/drug use, HIV status, and depression characteristics, as well as structural and social stressors and coping resources from the minority stress framework. Using linear regression, bivariate associations between structural stressors, social stressors, coping resources, and PHQ-2 sum scores were assessed. The multivariable linear regression model included all factors significantly associated with depressive symptoms at a level of *p* ≤ 0.10 in the bivariate analysis. Age, education, employment, and income were included as controls. Participants who were missing data on the outcome or covariates were excluded from the multivariable model. All analyses were completed in Stata v.13.

## 3. Results

### 3.1. Characteristics of Overall Sample

Almost all participants (92%) were born in El Salvador, Guatemala, Honduras or Nicaragua; two participants were born in Mexico and one participant was born in South America. The average age was 32.3 years (range = 22–50 years; std. dev. = 7.1). Only 18% of the sample had used illicit drugs other than marijuana in the past twelve months. Strikingly, 74% of the sample reported that they were currently employed.

One-third of the LTW in our sample (35%) were categorized as recently depressed (PHQ-2 ≥ 3), 47% experienced suicidal ideation at some point in their lifetime, and 32% had ever attempted suicide. Among those who reported suicidal ideation or attempted suicide, there were no significant differences in current depression (data not shown).

Overall, 32% of the participants were living with HIV. All participants except one correctly self-reported their HIV status; one participant self-reported being negative but received a positive result on the rapid test. Regarding HIV risk behavior, over 50% of LTW, regardless of documentation status, endorsed having exchanged sex at some point in their lifetime (data not shown). 

Lifetime experiences of psychological violence were nearly universally endorsed (95%), and 58% of the sample experienced psychological violence in the past twelve months. Physical violence experiences were common among participants, both over their lifetime (76%) and in the past twelve months (50%). Many participants (45%) experienced sexual violence in their lifetime and within the past twelve months (16%).

Among the undocumented group (*n* = 24; see Figure 1), there were four participants who never applied for legal immigration relief. All four reported that they were afraid of what might happen if their application was rejected, three reported that it was also too expensive, and three reported that they also did not have enough information about it. Among those who had applied for legal relief (*n* = 20), the majority had received legal aid through an HIV-specialty health clinic that includes in-house legal services and a Spanish-speaking attorney. Within the documented group (*n* = 14; see Figure 1), there were eleven participants who received immigration relief; they reported receiving legal assistance from private attorneys and non-governmental organizations that specialize in immigration law. 

### 3.2. Differences by Documentation Status

Table 1 describes and compares the groups by documentation status, along demographic and immigration characteristics, depressive symptoms, drug and alcohol use, HIV status, structural and social stressors, and coping resources. There were no significant differences between the two groups in terms of age, education level, employment status, or income. The undocumented group was more likely to be uninsured (30.0%) than the documented group (7.7%; χ^2^ = 6.46, *p* < 0.05). A higher proportion of the undocumented group reported being unstably housed at least once in the past twelve months (91.7%) compared to the documented group (64.3%; χ^2^ = 4.41, *p* < 0.05). 

In terms of immigration experiences, the two groups of participants were not statistically different (Table 1). No participants were ever deported to their country of origin (data not shown). At least half of the participants in both the documented and undocumented groups had ever been in immigration detention (Table 1). Among those who had ever been detained, one-third of the undocumented group experienced abuse by another detainee, and half of the documented group experienced abuse by a guard. A total of five participants across both groups reported that because of their immigration/documentation status, they stayed with a partner even when they wanted to end the relationship. 

There were significant differences in depression by documentation status (Table 1). Participants who were undocumented had significantly higher mean depressive symptoms scores on the PHQ-2 (2.7) than documented participants (1.4; *t* = 2.60, *p* < 0.05), and were more likely to be categorized as recently depressed (45.8% vs. 15.4%) (χ^2^ = 3.43, *p* < 0.10). Among the undocumented group 58.3% had ever been suicidal compared to 28.6% among the documented (χ^2^ = 3.14, *p* < 0.10).

There were no significant differences between the two groups in terms of drug and alcohol use or HIV status (Table 1). Any illicit drug use, including marijuana, was reported by 20.8% and 14.3% of the undocumented and documented groups, respectively (Table 1). Similarly, 75.0% of the undocumented and 57.1% of the documented participants met or exceeded the cutoff for hazardous drinking on the AUDIT-C. Among undocumented participants, 25.0% were HIV-positive, and among documented participants, 42.9% were HIV-positive. 

In terms of social stressors, LTW in the undocumented group were more likely to report experiences of employment discrimination in their lifetime (83.3%), compared to the documented group (57.1%; χ^2^ = 3.13, *p* < 0.10). The two groups were also significantly different on fear of deportation. There was a higher average fear of deportation score among the undocumented group (42.0), compared to the documented (30.3) (*t* = 2.50, *p* < 0.05).

Regarding experiences of violence, the proportion of participants who experienced psychological, physical, and sexual violence, were similarly high regardless of documentation status (Table 1). More than half of documented participants and more than three-quarters of undocumented participants reported ever experiencing physical violence. Among undocumented participants, 54.2% ever experienced sexual violence, compared to 28.6% of documented participants. More undocumented participants reported physical and sexual violence in the past twelve months, compared to the documented participants, but these differences did not reach statistical significance.

In terms of coping resources, there were no significant differences between the two groups with respect to social support from friends, family members, and significant others, as well as individual resilience. Both groups scored an average of about thirteen on the resilience measure (range: 6–18).

### 3.3. Factors Associated with Depressive Symptoms

Bivariate associations between depressive symptoms and demographic, structural stressor, social stressor, and coping resource variables are reported in Appendix A. Employment, income, documentation status, friends’ social support, resilience, and psychological violence were all significantly associated with depressive symptoms and thus, eligible for inclusion in the multivariable model. However, because nearly all participants had ever experienced psychological violence we decided not to include lifetime history of psychological violence in the multivariable model.

The multivariable linear regression results are reported in Table 2. All variables except for employment status persisted in being significantly associated with depressive symptoms. Being documented (vs. undocumented) (*β* = −1.48, *p* = 0.00) was inversely associated with depressive symptoms, as were other factors including: living above (vs. below) the federal poverty level (*β* = −1.75, *p* = 0.01); receiving more social support from friends (*β* = −0.24, *p* = 0.03); and higher levels of individual resilience (*β* = −0.19, *p* = 0.01). 

## 4. Discussion

This study found that past two week depressive symptoms were independently and significantly associated with immigration/documentation status (legal authorization to live/work in the U.S.) among a sample of immigrant, Spanish-speaking LTW. Guided by the minority stress framework, we identified documentation status as a structural stressor associated with depressive symptoms [4]. To date, clinical practice guidelines for working with transgender persons, informed by the minority stress framework, have not included recommendations for addressing documentation status [5]. Building on our findings, we provide suggestions for addressing documentation status in the context of health interventions for immigrant LTW.

The LTW in this study were unique from other studies in that they migrated from Central American countries with a recent history of civil war and were living in Washington, DC. Cities like Chicago and SF are over-represented among studies of transgender women and their Latinx communities have a much longer history of migration from Mexico [3]. Furthermore, participants in this study were surveyed in 2017, in the wake of significant anti-immigrant sentiment and policy in the U.S., a context associated with psychological distress among Latinx communities [30].

Unsurprisingly, the undocumented LTW in this study were less likely to have health insurance, and more likely to have experienced recent unstable housing than documented LTW. Undocumented immigrants lack access to services, including linguistically-compatible providers [31,32]. Lack of documentation status can thus serve as a barrier to LTW accessing services typically provided to other transgender women. For example, gender affirmation through legal name and gender marker change may not be available to undocumented LTW who do not have access to the legal system. In previous research, LTW noted that one gender affirming outcome of attaining asylum was the ability to legally change their name and gender marker [16]. At a minimum, service providers for LTW should be aware of documentation status as a structural stressor and provide options to apply for legal relief, if these options are available [33].

Health interventions could minimally provide information to undocumented immigrant LTW on immigration-related legal options, such as asylum and U-Visas [13,34]. Interested immigrant LTW could receive referrals to attorneys for more extensive screening regarding eligibility for immigration relief. These attorneys should receive training in working with transgender communities, ideally speak Spanish, and be sensitive to issues of fear of deportation and discrimination due to gender identity.

While some immigrant LTW may be reluctant to seek legal services, others may be motivated by the benefits of receiving immigration-related legal relief, including having a social security number, health insurance, and a legal work permit [16]. The majority of LTW participants in this study either received or were in the process of applying for legal relief. Their pursuit of legal relief was likely due to referrals by active CBOs and a local, favorable immigration court. Applying for immigration legal relief may be less attractive or more risky for immigrant LTW living in areas of the U.S. where the local immigration court has a lower asylum granting rate. In this case, legal providers could inform LTW about the diversity in asylum granting rates by geographic location. 

In terms of other social stressors under the minority stress framework, this study did not find an association between depressive symptoms and experiences of violence (lifetime or recent). A previous study among LTW found an association between lifetime experience of sexual violence and depressive symptoms [6]. However, it is notable that violence was highly prevalent for the LTW who participated in this study. Three-quarters of participants experienced physical violence by any perpetrator in their lifetime. It may be that violence was more normative among this study’s LTW participants because they migrated from historically violent Central American countries. Violence towards transgender people is particularly high in Latin American countries. In 2009, Latin American countries accounted for 88% of all murders of transgender people in the world [35]. Future research with immigrant LTW should include temporal measures that account for occurrences of violence in country of origin and country of residence.

LTW in this study also reported high rates of recent experiences of violence in the U.S. Half of the LTW experienced physical violence in the past twelve months. These high levels of recent violence are consistent with discriminatory violence that occurs towards transgender people in the U.S. For example, in the U.S. transgender survey, 12% of LTW surveyed were physically attacked in the past year because of being transgender, and 54% had ever experienced violence by an intimate partner [36]. We do not know whether our participants’ recent experiences of violence were associated with an increase in anti-immigrant sentiment; this is a question for future research.

In terms of other social stressors, we did not find evidence that employment discrimination was associated with depressive symptoms among immigrant LTW. However, a higher proportion of the undocumented group experienced employment discrimination in their lifetime. Research has described how undocumented LTW experience discrimination or harassment from their employers and have no legal recourse [16]. After garnering asylum, some immigrant LTW reported securing better and less discriminatory employment [16]. It may be that there is an indirect effect of documentation status on mental health through employment discrimination. 

Similarly, fear of deportation, another social stressor, was not associated with depressive symptoms, and may be a consequence of the effect of documentation status. Using a new and psychometrically sound scale measuring fear of deportation that was developed in Spanish, we found that the undocumented LTW reported higher levels of fear of deportation. For LTW, fear of deportation may be exacerbated by fear of returning to their countries of origin, because of profound violence and discrimination experienced there [16]. While violence certainly occurs towards transgender people in the U.S., there are also safe spaces for transgender communities in the U.S. that are not generally found in Central America.

This study’s findings corroborate previous research demonstrating that income and social support are associated with depressive symptoms among LTW [6]. The minority stress framework underscored the importance of social support as protective against minority stress, and research specific to transgender populations has demonstrated the protective role of social support against depression for LTW [6]. We found that friends’ support, not support from families or significant others, was associated with reduced depressive symptoms. As others have suggested, it may be that LTW have a particular need for friends’ support because they separated from families during migration [6] or because they experienced discrimination by their families [16]. Interventions for immigrant LTW could thus focus on strengthening relations with and support from friends. Consistent with the minority stress framework, our study also found resilience to be a protective factor against depressive symptoms for immigrant LTW, suggesting that empowerment interventions that enhance resilience might prove fruitful for this population.

## 5. Limitations

While there were a number of strengths to our study, including the unique sample and the inclusion of new measures, there are limitations to consider. Given the cross-sectional nature of our study, we are not able to assess causal claims. However, we measured contemporaneous depressive symptoms and current, not historical, legal status, which is a strength. The small sample size and use of convenience sampling also hampers our ability to draw generalizations about our findings. Although we included two proximate cities to try to attain a larger sample, all the immigrant LTW lived in DC. This may be because there is no CBO specifically serving Latinx sexual and gender minorities in Baltimore, while there are a few in DC. We also did not measure or control for gender dysphoria, which can contribute to depressive symptoms among transgender people. Finally, we would have liked to know whether any of our participants specifically attributed their depressive symptoms to their immigration status, and we did not assess this perception. However, previous qualitative research among LTW pointed to the positive effects that receiving legal immigration relief had on gender identity expression, access to services, attainment of less risky employment, and control over sexual relationships [16]. 

## 6. Conclusions

In this paper, we highlight immigration/documentation status as an important correlate of depressive symptoms for LTW. Using novel measures and a mostly Central American, non-Mexican sample of LTW, we found that having legal immigration documentation was protective against depressive symptoms for immigrant LTW. Given that immigration status is a structural determinant of health, and that there has been a call for structural interventions to address HIV and mental health among transgender women of color [37], immigration status should be a focus of future research. The majority of the participants in our study received legal aid at a health clinic involved in a medical legal partnership [38]. Thus, medical legal partnerships are also a promising approach for addressing both immigration status and healthcare needs of LTW.

## Figures and Tables

**Table 1:** Characteristics and Experiences of Immigrant Latina Transgender Women (*n* = 38) in the STROBE Study, Washington, DC, USA, 2017.

	Current Documentation Status		
	Undocumented (*n* = 24) % (n) or Mean (SD)	Documented (*n* = 14) % (n) or Mean (SD)	*t* or χ^2^
**Demographic Characteristics**			
Age, years (range: 22–50)	32.1 (7.5)	32.8 (6.6)	−0.29
Highest Education Level			1.97
Less than high school	54.2 (13)	50.0 (7)	
High school	20.8 (5)	35.7 (5)	
Some college	16.7 (4)	14.3 (2)	
Bachelor’s degree or higher	8.3 (2)	0.0 (0)	
Unemployed	20.8 (5)	35.7 (5)	1.01
Income below federal poverty level	91.3 (21)	76.9 (10)	1.44
Health Insurance			6.46 **
Uninsured	30.0 (6)	7.7 (1)	
Public insurance	70.0 (14)	69.2 (9)	
Private insurance	0.0 (0)	9.1 (3)	
Unstable housing in previous 12 months	91.7 (22)	64.3 (9)	4.41 **
**Immigration Experiences**			
Born in Central America	100.0 (24)	93.0 (13)	
Ever been in immigration detention	50.0 (12)	57.1 (8)	0.18
Among those detained, experienced abuse by another detainee	33.3 (4)	12.5 (1)	1.11
Among those detained, experienced abuse by a guard	25.0 (3)	50.0 (4)	1.32
Because of immigration status, stayed with a partner even when wanted to end the relationship	16.7 (4)	7.1 (1)	0.70
Type of Immigration Relief Applied For	*n* = 20	*n* = 11	3.16
Asylum	90.0 (18)	63.6 (7)	
U-Visa	5.0 (1)	18.2 (2)	
Temporary protected status (TPS)	5.0 (1)	18.2 (2)	
**Depressive Symptoms**			
PHQ-2 sum (score range: 0–6)	2.7 (1.6)	1.4 (1.2)	2.60 **
Meets criterion for depression (PHQ-2 > 3)	45.8 (11)	15.4 (2)	3.43 *
Lifetime suicidality	58.3 (14)	28.6 (4)	3.14 *
**Drug and Alcohol Use**			
Drug use past 12 months			1.41
Illicit drug use	20.8 (5)	14.3 (2)	
Marijuana only	8.3 (2)	21.4 (3)	
No drug use	70.8 (17)	64.3 (9)	
Audit-C (binary)	75.0 (18)	57.1 (8)	1.31
**HIV Status**			
HIV-positive test result (OraQuick)	25.0 (6)	42.9 (6)	1.30
**Social Stressors**			
Employment discrimination in lifetime	83.3 (20)	57.1 (8)	3.13 *
Fear of deportation sum (score range: 16–64)	42.0 (13.7)	30.3 (12.3)	2.50 **
Lifetime psychological violence	95.8 (23)	92.9 (13)	0.16
Lifetime physical violence	83.3 (20)	64.3 (9)	1.77
Lifetime sexual violence	54.2 (13)	28.6 (4)	2.34
Psychological violence, past 12 months	58.3 (14)	57.1 (8)	0.01
Physical violence, past 12 months	58.3 (14)	35.7 (5)	1.81
Sexual violence, past 12 months	16.7 (4)	14.3 (2)	0.04
**Coping Resources**			
Friends’ social support (range: 4–12)	10.7 (2.5)	11.1 (1.5)	−0.64
Family social support (range: 4–12)	10.0 (2.7)	8.3 (3.5)	1.71 *
Significant other social support (range: 4–12)	11.3 (2.0)	11.1 (2.2)	0.25
Resilience (range: 6–18)	13.0 (3.4)	13.4 (3.9)	−0.33

* *p* < 0.10; ** *p* < 0.05.

**Table 2:** Multiple Regression Analysis on Recent Depressive Symptoms among Immigrant Latina Transgender Women in the STROBE Study, Washington, DC, USA, 2017 (*n* = 35).

Variables	Reference Group	Standardized B	Std Error	*t*	*p*-Value
Age		−0.03	0.03	−0.94	0.35
Employed	Unemployed	−0.66	0.56	−1.19	0.24
Highest education level	Less than high school	−0.17	0.21	−0.08	0.93
Income	Below federal poverty level	−1.75	0.58	−3.01	0.01
Documentation status	Undocumented	−1.48	0.41	−3.59	0.00
Social support from friends		−0.24	0.11	−2.27	0.03
Resilience total		−0.19	0.06	−3.05	0.01

Note. *R*^2^ = 0.65, F_7,27_ = 7.23, *p* < 0.01.

## Appendix A

**Table A1:** Bivariate Associations with Depressive Symptoms among Immigrant Latina Transgender Women in the STROBE Study, Washington, DC, USA, 2017.

Variables	Reference Group	Standardized B	Std. Error	*t*	*p*-Value *
**Demographic Characteristics**					
Age, years		0.00	0.04	0.02	0.98
Highest education level	Less than high school	−0.10	0.29	−0.34	0.74
Employment	Unemployed	−1.04	0.60	−1.73	**0.09**
Income	Below federal poverty level	−1.17	0.78	−1.50	0.14
Health insurance	Uninsured	−0.11	0.50	−0.21	0.83
**Structural Stressors**					
Documentation status	Undocumented	−1.28	0.52	−2.49	**0.02**
Housing in previous 12 months	Unstable	0.80	0.67	1.19	0.24
**Social Stressors**					
Employment discrimination, lifetime	No discrimination	0.87	0.60	1.44	0.16
Fear of deportation		0.02	0.02	0.96	0.34
Lifetime psychological violence	No psychological violence ever	2.34	1.11	2.11	**0.04**
Lifetime physical violence	No physical violence ever	0.58	0.61	0.94	0.35
Lifetime sexual violence	No sexual violence ever	0.06	0.54	0.11	0.91
Psychological violence, past 12 months	Experienced violence	0.49	0.53	0.92	0.36
Physical violence, past 12 months	Experienced violence	−0.20	0.53	−0.38	0.70
Sexual violence, past 12 months	Experienced violence	0.34	0.72	0.47	0.64
**Coping Resources**					
Friends’ social support		−0.27	0.11	−2.38	**0.02**
Family social support		0.04	0.09	0.44	0.66
Significant other social support		0.00	0.13	0.03	0.97
Resilience		−0.25	0.07	−3.74	**0.00**

* bolded values are significant for inclusion at a level of α = 0.10 in the multivariable model.
