# Erratum: Yamanis, T., et al. Legal Immigration Status is Associated with Depressive Symptoms among Latina Transgender Women in Washington, DC. *Int. J. Environ. Res. Public Health* 2018, *15*, 1246

**DOI:** 10.3390/ijerph15122619

**Published:** 2018-11-22

**Authors:** Thespina Yamanis, Mannat Malik, Ana María del Río-González, Andrea L. Wirtz, Erin Cooney, Maren Lujan, Ruby Corado, Tonia Poteat

**Affiliations:** 1 School of International Service, American University, 4400 Massachusetts Ave NW, Washington, DC 20016, USA; maren.lujan@gmail.com; 2 Bloomberg School of Public Health, Johns Hopkins University, Baltimore, MD 21205, USA; mmalik7@jhmi.edu (M.M.); awirtz1@jhu.edu (A.L.W.); ecooney2@jhmi.edu (E.C.); tpoteat@jhu.edu (T.P.); 3 Department of Psychology, The George Washington University, 2121 I St NW, Washington, DC 20052, USA; amdelrio@gwmail.gwu.edu; 4 Casa Ruby, 7530 Georgia Ave NW, Washington, DC 20012, USA; corado@casaruby.org

The authors would like to update the “Acknowledgements” section of their previous paper [1] as follows: 

## References

[1] Yamanis T., Malik M., del Río-González A.M., Wirtz A.L., Cooney E., Lujan M., Corado R., Poteat T. (2018). Legal Immigration Status is Associated with Depressive Symptoms among Latina Transgender Women in Washington, DC. Int. J. Environ. Res. Public Health.

